# The Analysis of Residents’ Intention to Consume Pre-Made Dishes in China: A Grounded Theory

**DOI:** 10.3390/foods12203798

**Published:** 2023-10-17

**Authors:** Yanling Xiong, Xiaoxi Lin, Xiaowei Wen, Yiqin Wang, Wenwen Liang, Tianyang Xing

**Affiliations:** 1 College of Economics and Management, South China Agricultural University, Wushan Road 483, Tianhe District, Guangzhou 510642, China; 2 Research Institute of Rural Development of Guangdong Province, South China Agricultural University, Wushan Road 483, Tianhe District, Guangzhou 510642, China

**Keywords:** pre-made dishes, grounded theory, consumer motives, consumer intention

## Abstract

The demand for pre-made dishes has increased in China. However, a detailed understanding of residents’ intention to consume pre-made dishes is lacking in the existing studies. This research aims to investigate the consumer motives and intention to consume pre-made dishes. Through in-depth interviews and analysis, this research explores the factors influencing the residents’ intention along the research steps of grounded theory. Fifty-one residents participated in semi-structured interviews via face-to-face or online interviews. Three motives for purchasing pre-made dishes were attitude, subjective norm, and perceived control. These were influenced by external factors, including environmental features and product features. Subjective characteristics are pre-existing characteristics of individuals themselves, including cooking skills, food skills, housework allocation, and eating attitudes, which play a moderating role in the relationship between external factors and consumer motives. Based on the six major categories, this study built a model of the formation mechanism of the consumer intention to consume pre-made dishes. It revealed the psychological attribution of residents’ consumption of pre-made dishes. The finding of this study contributes to the understanding of the internal logic of PMDs’ consumer intention formation. It would be a guide for researchers to map out appropriate business development strategies, and provide evidence for the government in formulating management policies.

## 1. Introduction

**Figure 1:**
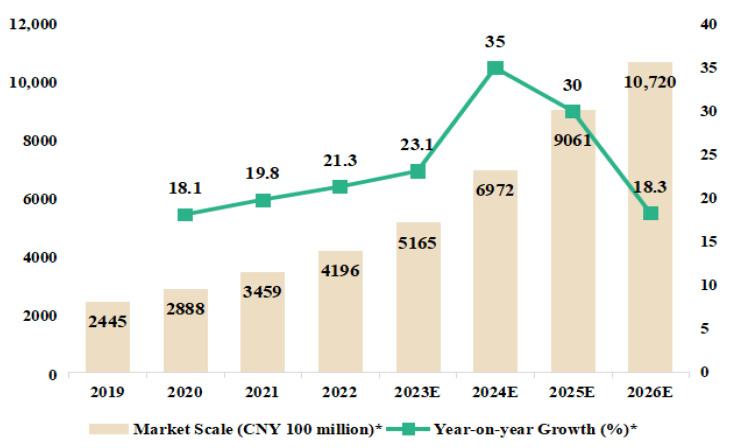
Market scale and forecast of China’s pre-made dishes industry from 2019 to 2026. E: Expected. * Data were collected online from iiMedia Research in March 2023.

In a busy world, consumers spend less time on food shopping and preparation. As a result, the convenience food sector has seen rapid growth across the world [1,2]. The global convenience food market is expected to reach an impressive total revenue of USD 1126.8 billion by the end of 2025 [2]. Focusing on the European convenience food market, the sales turnover of ready meals and soups has shown a consistent increase over the past years in Germany. It increased from EUR 6.3 million in 2012 to EUR 7.3 million in 2019, and peaked at EUR 8.6 million in 2020 [3]. As for the market in Oceania, the Australian ready-meals market has experienced an average annual increase of 8% since 2014 [4]. In the Asia–Pacific region, Asians tend to pursue convenience in their traditional diet. The Korean home meal replacement (HMR) market grew by an average of 15.2% annually, reaching KRW 4 trillion in 2020 [5]. In China, the pre-made dishes (PMDs) have also gained in popularity over recent years, due to social factors such as shrinking household sizes and a fast-paced lifestyle. Socializing housework has become a trend [6]. To control the epidemic spread, restrictions on private gatherings and establishing a quarantine at home were recommended in the past few years, which further increased the frequency of family meal scenarios and accelerated the growth of the PMDs market [7]. Simultaneously, the Chinese government has actively encouraged food manufacturers and supermarkets to expand the PMDs market. The supply and distribution channels for PMDs products have been improved in recent years, which enhanced the shopping experience for consumers, and further fueled their demand and desire to purchase PMDs products. Additionally, in a significant milestone, the “No. 1 central document” in 2023 officially included PMDs products, “improving the standardization and standardization level of industries such as clean dishes and central kitchens, and cultivating and developing the PMDs industry” [8]. According to statistics, the Chinese PMDs market size reached CNY 415.15 billion in 2022, with 64,000 existing PMDs enterprises [9]. Sales forecasts indicate that the PMDs market will continue to rise at a high growth rate of around 20% annually within the next three to five years. It is projected to be a staggering CNY 1072 billion by the year 2026 (Figure 1) [10,11]. This indicates that the PMDs industry holds tremendous potential for further development in China.

PMDs are made from edible products from agriculture, forestry, animal husbandry, and fisheries, with or without a variety of auxiliary ingredients and food additives. They are designed to transfer the time and activities of preparation from the household to the food processor for a fast lifestyle [12,13]. In general, according to the cooking and preparation process, the products can be classified into four types: ready to eat (RTE), ready to heat (RTH), ready to cook (RTC), and ready to prepare (RTP). Considering the degree of product completion, the PMDs can also be divided into primary products, semi-finished products, and finished foods [5,14,15]. To conform to Chinese dietary habits, PMDs cover a wide range of original products, and give residents free rein in cooking. They are often more convenient and easier to manage than home cooking, since PMDs just need to be heated up and cooked with ready vegetables, meats, and sauces. Meanwhile, they assist people in releasing more time and energy inputs to be utilized in paid work outside the home, especially for women [16,17]. Although convenience food was criticized for the negative burden imposed on the environment compared to home-making [18], it can be mitigated in production phases both by mandatory and voluntary standards [19]. In fact, the majority of fresh local produce is highly fermentable and perishable. However, this issue can be effectively addressed through food processing, which not only benefits the environment but also enables the development of strategies for reuse, thereby adding economic value and promoting valorization [20,21]. Additionally, compared to other countries the emission of volatile organic compounds (VOCs) is higher in China due to the diverse eating habits and cooking styles. VOCs in cooking oil fumes, the third-largest source of urban air pollution, can be detrimental to both the environment and human health. Nevertheless, the utilization of PMDs can substantially reduce the concentration of kitchen oil fumes [22], addressing the environmental and health concerns.

Traditional viewpoints suggest that, as a kind of convenience food, PMDs are often considered unhealthy, unsustainable, and more processed, and, relatedly, a less acceptable way of providing food for the family [23,24]. However, an increasing number of people have started purchasing PMDs. The research on the reasons behind this phenomenon is scarce. In addition, according to previous studies PMDs have been referred to as “home meal replacements (HMRs)”, “convenience food”, “instant food”, and “prepared meals”, depending on the content and purpose of the study [5,14]. Research has found that there are various factors that consumers consider while making purchase decisions of convenience food, such as brand, ease of cooking, food quality, reasonable price, accessibility, and quality of delivery service [25,26,27,28,29]. Research has also shown that demographic characteristics, such as age, income, educational attainment, and full-time employment, would influence residents’ choice of HMRs. For instance, compared to couples and families with children, single-person households, especially single men, tend to consume HMRs [6,30]. Given the many similarities between HMRs and PMDs, it is an effective method to refer to these studies to understand residents’ purchase behavior toward PMDs products. However, the previous studies still have some shortcomings as they often focus on certain factors, and have not systematically sorted out the influences that affect consumer motives and willingness to consume PMDs.

Moreover, several research projects have studied convenience food consumption by applying the Theory of Planned Behavior (TPB) and Structural Equation Models (SEMs) to elucidate the influencing factors on consumer behavior [31,32,33]. However, a SEM is a typical method used to test complete theories and concepts [34], and there is a dearth of research investigating consumer motives and intentions without preconceived assumptions. Additionally, it is important to note that these studies, which are primarily based on research conducted in other countries, may possess a diminished explanatory power when applied within the Chinese context [35]. It can be seen that few frameworks have been developed to understand the PMDs purchase intention formation process at present, and studies on residents in China are still insufficient.

To our knowledge, existing studies have not systematically examined the formation of PMDs’ consumption motives and intention, and have not delved into the underlying mechanisms of action. Hence, the present study aims to contribute to the current body of literature by studying the relationships between the motives and intention through the use of grounded theory. Firstly, our study investigates the internal and external factors that influence PMDs’ consumer motives and intention. Secondly, it constructs a theoretical model including the factors that drive consumer intention toward PMDs to show the mechanism. Thirdly, this study provides insights into the mechanism motivating residents to purchase PMDs products for government and PMDs companies. By fulfilling these objectives, this study aims to contribute valuable knowledge to the field of PMDs, enabling stakeholders to make informed decisions and effectively cater to consumer needs and preferences in the market.

## 2. Methods

To achieve the research objectives, this study adopted grounded theory, which is a qualitative research methodology often used to develop an emerging theory induced by the raw data and empirical material [36]. Unlike quantitative methods, qualitative methods focus on understanding the analyzed phenomenon through the perspective of its participants. These methods evoke a narrative or analytical richness, which brings out more detail and nuance from a case than can be found by reducing it to quantitative measures [37,38,39]. They are widely used in various social science disciplines, such as sociology, anthropology, psychology, and education, to delve into human behavior, attitudes, and experiences [39]. Thus, prior to quantitative methods, applying qualitative methods is more suitable for this study. Grounded theory is used for this study as it seeks to develop a theoretical understanding of the analyzed phenomenon based on the experiences of the participants [40]. It advocates avoiding preconceived assumptions, and emphasizes that the formulation of research questions and the development of theories is a natural emergent process [41]. It is also applicable to factor identification and process interpretation, and is often used to analyze exploratory questions on new topics that are not easy to grasp [42]. Researchers can identify core concepts and construct theories through the analysis of empirical data. This is consistent with the objective of this study.

### 2.1. Sampling and Participant Recruitment

To guarantee the accuracy of the sample and obtain extensive information, the participants consisted of residents from diverse age groups, occupations, and educational backgrounds in China. They were recruited both online and in person. Then, the research group randomly selected several subjects to check and correct the interview questionnaire logic. Based on the pilot test, the research group conducted a theoretical sampling. The determination of the sample size was based on the principle of theoretical saturation. When no new meaningful information was being generated, data collection was stopped [37,43,44]. A total of fifty-one residents were ultimately selected to participate in this study. Among them, forty-five interview samples were randomly selected as formal coding material, and the remaining six interview samples were used for saturation testing.

The fifty-one semi-structured interviews were conducted by the first author between December 2022 and March 2023, including twenty face-to-face interviews and thirty-one online interviews. The first author collected residents’ cell phone numbers upon their agreement to participate and sent out an interview recruitment notification via SMS. The interviews lasted approximately 15 to 50 min and were audio-recorded with the consent of the participants. At the end of the interviews, each participant was compensated with CNY 20 (USD 2.79) in cash. This study was approved by the Survey and Behavioral Research Ethics Committee at South China Agricultural University. All participants were fully informed of the purpose of this study and provided informed consent.

### 2.2. Interview Study Design

Interview questions in the present study were developed to understand residents’ perspectives on PMDs products. The interview guide was divided into three sections (Appendix A): (1) demographic information of the participants (e.g., gender, age, career, and education level), (2) participants’ perceptions of home cooking (e.g., the way to purchase food material, arrangements for household chores related to cooking, the skills related to home cooking, and the advantage and disadvantage of home cooking), and (3) participants’ perceptions of PMDs (e.g., cognitive and affective attitudes toward PMDs, planning and shopping for the pre-made dishes, and preferences and expectations for PMDs products).

### 2.3. Data Analysis

**Figure 2:**
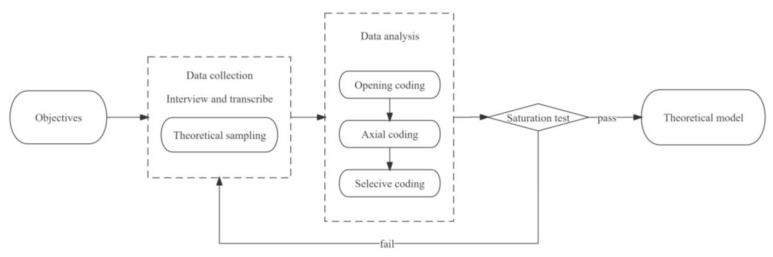
The framework of the research process.

The basic information about the PMDs and transcripts of the interview recordings totaling more than 120,000 words were included as raw data for the grounded theory-based analysis. This study strictly followed the grounded theory research framework of developing abstract concepts and theories through a top-down approach. This study’s coding methods included open coding, axial coding, and selective coding [44]. Firstly, two trained researchers transcribed and checked the interview recordings. Secondly, the transcripts were coded and analyzed in Nvivo12 Plus, a qualitative analysis software designed by QSR International Proprietary Limited. Two researchers coded independently and compared codes with each other to ensure consistency and reliability. After discussion, an initial codebook was finalized. Thirdly, two researchers coded all transcripts based on the initial codebook separately, and then compared and discussed each code. Fourthly, similar codes were grouped into the same categories, and the core categories were extracted from these categories [45,46]. Finally, when there were no new categories, new relations between categories, or new properties of each category, core categories reached theoretical saturation and a theoretical model was constructed. The research process of this study is shown in Figure 2.

## 3. Results

### 3.1. Demographic Profile of Participants

A total of 51 residents participated in the semi-structured interviews (Table 1). Most of them were female (n = 32), married people, and aged between 25 and 54 years. Around sixty-nine percent of the participants had a bachelor’s degree or higher. To gain more perspective, the participants were from all walks of life, and the respondents came from several regions of China. According to IiMedia Research’s White Paper, among consumers of PMDs, female consumers account for 62.1%, while male consumers account for 37.9% in China. It is noteworthy that 80.6% of consumers are married, and about 68.8% of these people are both married and parenting. Furthermore, individuals aged between 22 and 40 constitute 85.2% of the PMDs spenders, especially those people who live in first- and second-tier cities [11]. It can be seen that our sample selection is representative and reasonable.

**Figure 3:**
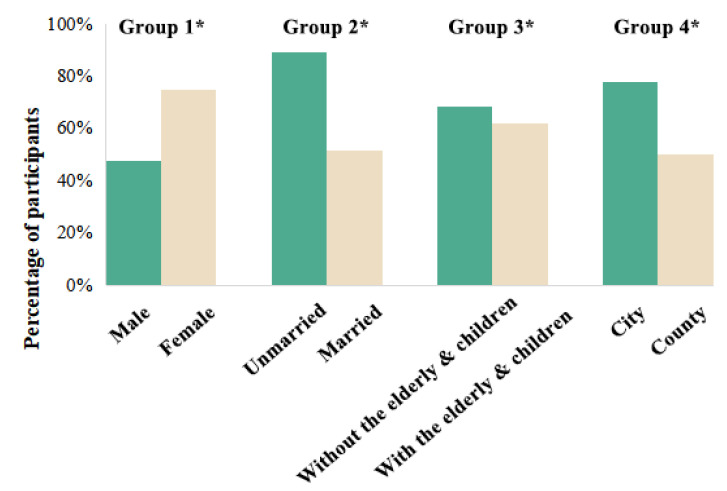
Purchase intention of PMDs based on responses from 51 participants. * The percentage of participants who responded “yes” to the question of PMDs purchase intention, e.g., Group 1: Male = male (answer yes)/total male × 100%; Female = female (answer yes)/total female × 100%.

**Figure 4:**
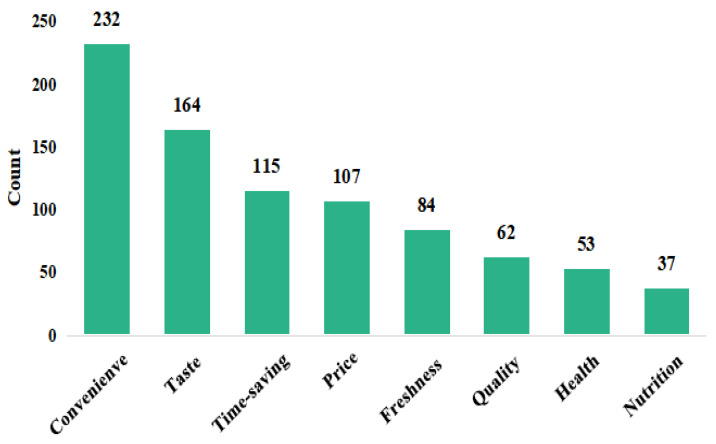
Word-frequency statistical chart.

Figure 3 shows the survey results of PMDs’ consumer intention. More females were willing to purchase PMDs than males (Group 1). Compared to people who have been married, single people tended to buy PMDs for saving time and energy (Group 2). Considering the health of the elderly and children, households without the elderly and children were more likely to purchase PMDs products than households with the elderly and children (Group 3). Due to a fast-paced lifestyle and abundant resources, people living in the city were more likely to consume PMDs (Group 4). The results are consistent with previous studies. Additionally, this paper filtered out eight points that participants frequently mentioned when making choices in the face of PMDs (Figure 4). The results showed that their major concern was convenience. The biggest concerns were taste, time-saving, price, and freshness. To summarize the driving factors of consumer intention, we use grounded theory for further analysis.

### 3.2. Open Coding

Opening coding is to code original transcripts word-by-word, which aims at identifying phenomena, abstract labeling, and defining concepts. Gradually, the relevant concepts and categories were proposed. To restore the meanings expressed by participants as closely as possible, this study used many original words of the participants to name labels. The coding process included labeling, conceptualization, and categorization. For instance, first, the participants’ original statements were labeled as “Shredding is difficult for me”, “I can cook simple dishes”, and “I do not know much about how to handle some ingredients”. Then, the above labels were summarized to form the initial concepts of “the level of cooking skills” and “ability to prepare food”. Finally, the three concepts were categorized as “cooking skills”. After several stages of generalization, comparisons, and corrections, 70 initial concepts were refined, leading to the final refinement of 21 categories (see Table 2).

### 3.3. Axial Coding

Based on the initial concepts and categories, axial coding aims to explore and construct relationships among the concepts, sub-categories, and main categories [44]. Through repeated study and cluster analysis of the concepts, the highly abstract concepts (main categories) were developed. To reveal the causal relationships among the concepts, sub-categories, and main categories, this study investigated initial concepts and hidden contexts with the help of Nvivo12 plus. Referring to behavioral reasoning theory [47], four main categories were abstracted, including internal reasons, external reasons, and consumer motives and intention (Table 3). This process of axial coding allowed for a deeper understanding of the interconnections and influences that shape residents’ perspectives on PMDs consumption.

### 3.4. Selective Coding

Selective coding, which can also be referred to as core coding, aims to identify core categories. Through a systematic analysis, a core category was discovered, and a “storyline” was formed to connect the core category with other categories [48]. The “storyline” not only systematically organizes the relationships between categories, but also encompasses a variety of phenomena and venation. The typical relationship structure and representative statements are shown in Table 4. This approach enhances the coherence and comprehensiveness of the findings, leading to a deeper understanding of the underlying patterns and connections among the identified categories.

#### 3.4.1. External Reasons → Consumer Motives

External reasons include the factors that residents consider when attempting to purchase PMDs products. According to axial coding, the factors were divided into environmental features and product features. Environmental features consist of the factors that could provide information about PMDs, and product features refer to the diverse attributes of PMDs that residents perceive. Residents’ consumer motives were affected by the external reasons. Specifically, factors such as advertisement, brand, packaging, price, or quality influenced residents’ cognitive and affective attitudes. And, they also perceived the value and risk of PMDs through these factors. These can be observed from the following participant quotes.

Twenty-nine participants indicated that they recognized and learned about PMDs through environmental features such as advertisements, self-published media, friends and relatives around them, and word-of-mouth.

A17: We are certainly seeing a lot of hype by PMDs companies, such as notifications in subscriptions on the Chinese social network WeChat. The concept of PMDs has managed to attract significant public attention over time. You will definitely be affected by these. (Obtained information about PMDs from the media).

A37: Because some people just say this dish is delicious, and then I am going to look for feedback on the product and try to buy some. (The curiosity was aroused by word-of-mouth).

Participants were also concerned about various attributes of PMD products, such as flavor, freshness, texture, branding, packaging, and other aspects of the products.

A21: I prefer brand-name PMDs products, since they are more reliable. And I would also choose the products with moderate price or maybe a little higher price. I feel relieved with such products. (Pay attention to brand and price).

#### 3.4.2. Consumer Motives → Purchase Intention

Consumer motives are the desire that drives consumers to engage in purchases, including subjective norm, attitude, and perceived control. Subjective norm represents the pressure that residents perceive from important others. Attitude means residents’ rational and emotional evaluation of the behavior. Perceived control implies the uncertainty about consumer purchasing decisions. For instance, when important others do not support PMDs purchases, it would diminish residents’ desire to consume. A positive attitude toward PMDs would be actively considered by residents. The same situation happens when they perceive values from the behavior. Therefore, consumer intention was influenced by consumer motives, as the following extracts show.

First, as for subjective norm, eleven participants expressed that other individuals’ behaviors and attitudes would affect their intention, and fourteen participants said that they would conform to the expectations of others.

A09: I believe I am being influenced by my colleagues, as a considerable number of them are single and do not have to worry about cooking. They often bring PMDs to the office. Therefore, I will also buy them and share them with my colleagues. (Affected by other individuals’ behaviors and attitudes in the social reference group).

A27: My parents think PMDs are very unhealthy and do not allow me to buy them. The elders definitely cannot accept such products and I would not buy PMDs when they are at home. (Be obedient to others’ expectations).

Second, cognitive and affective attitudes positively influenced consumer intention. Thirty-one participants mentioned that they would purchase PMDs products when they have positive attitudes toward the products.

A02: Anyway, the attitude is “I do not want to buy it, since I know nothing about it”. (A low level of knowledge hinders willingness to buy PMDs).

A24: I would worry about the quality and safety of PMDs, and I think they are unhealthy, unhygienic, and un-nutritious. I might not buy them. (Negative attitudes hinder willingness to buy PMDs).

Third, measuring the perceived value of a good or service is a necessary mental process for consumers before making consumption decisions. Interview data suggested that a high perceived value induces a higher willingness to consume, while a high perceived risk induces a lower willingness to consume.

A03: As a processing product, the PMDs might hide unpleasant smells and tastes through processing technology. It is feared that the PMDs might use leftover material, which is not good and fresh. Then I would not buy them for the reason that it might cause a food safety risk. (Perceived risk reduces willingness to buy PMDs).

#### 3.4.3. Internal Reasons’ Moderation

Subject characteristics represent skills and attitudes involved in home cooking. Interview data showed that internal reasons moderate the effects of external reasons on consumer motives. When the moderating effect of internal reasons is weak, the influence of external reasons on motives gets stronger, whereas the influence becomes weaker when their effects are stronger. For example, individuals with good cooking skills are more likely to purchase PMDs. The participants’ quotes are shown below.

A25: I am not good at cooking. I could be influenced by live streaming to buy pre-made dishes. For example, when you are in live streaming chat rooms, the hosts of live streaming chat rooms will describe the products and other information, such as how long it takes to cook it. These will prompt you to place an order.

A50: As a professional chef, I feel that cooking, washing and chopping are easy. I am very experienced in cooking. I will not buy these things. However, for people who are not professional chefs, they may buy PMDs. (Subject characteristics moderate the relationship between external reasons and consumer motives).

#### 3.4.4. Consumer Motives’ Mediation

The data suggested that external reasons affect consumer intention via consumer motives, namely subjective norm, attitude, and perceived control, which act as mediators. First, through the interaction of information, residents perceive values and risks, and then this would influence consumer intention. Second, external reasons would also affect important others’ attitudes, and then residents might perceive social pressure through interpersonal direct interaction. Ultimately, consumer intention would be influenced. Third, the various information about PMDs can stimulate consumers’ cognitive and affective attitudes, thereby influencing their willingness to consume. These can be evidenced by the following extracts.

A25: In the live broadcast, they will present products and explain the taste to you. And they will tell you how long it takes to make it. This information will motivate you to buy the products. (The publicity raises the level of knowledge, which promotes the willingness to purchase PMDs products).

A27: PMD is a kind of “junk food”. My parents think it is very unhealthy, and they do not allow me to buy it. I will not buy it, especially at home. (Product’s features negatively affect parents’ attitudes, which reduce the willingness to purchase PMDs products via subjective norm).

A34: If the products are too cheap, I will worry about the quality of the products. Because the price was set based on the cost. In addition, I also pay attention to product packages. The packages should be attractive. Therefore, I prefer to buy products with proper prices and good packages. (Price and package positively affect perceived risks, which promotes the willingness to purchase PMDs products).

**Figure 5:**
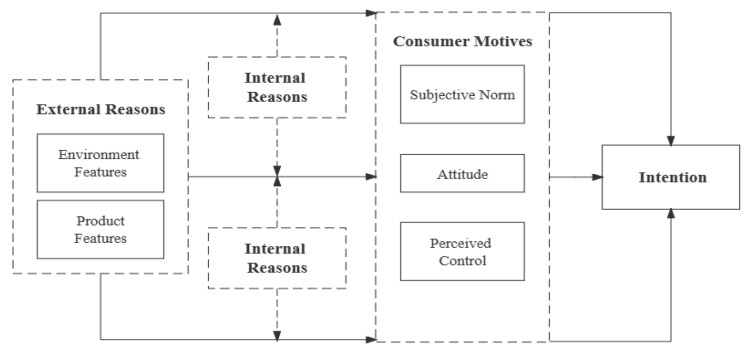
The theoretical model of the influencing factors of PMDs’ consumer intention.

Based on the above analysis, a complete theoretical model was developed. In this study, the core category is “the mechanism for the formation of intention to consume PMDs”. Specifically, the factors influencing PMDs’ purchase intention can be categorized into the following three main categories: external reasons, internal reasons, and consumer motives. The “storyline” can be summarized as follows. External reasons, including environmental features and product features, are factors leading to PMDs’ consumer motives (subjective norm, attitude, and perceived control). External reasons are predisposing factors of PMDs’ consumer intention, which influence consumer motives, thereby propelling consumer intention. Internal reasons (subject characteristics) can moderate the relationship between external reasons and consumer motives. There is a reciprocal relationship between external reasons and internal reasons. When external reasons significantly affect consumer motives, internal reasons’ moderating effect is not obvious. On the contrary, the moderation effect is significant when external reasons have a weak impact on consumer motives. The theoretical model of the influencing factors of PMDs’ consumer intention is illustrated in Figure 5.

### 3.5. Categories’ Saturation Test

According to previous studies, this study ultimately collected 51 samples, completely fulfilling the standard of the sample size [49,50]. To fully identify the influencing factors of PMDs’ consumer intention, the two researchers used the remaining six interview samples to check whether there are new categories. The results indicate that the test results remained aligned with the factors identified in previous analyses, unveiling no novel connections or classifications [40]. Theoretical saturation was determined.

## 4. Discussion

By integrating the findings of this study with existing research, a comprehensive theoretical model of the determinants of consumer intention toward PMDs was developed. This model provides valuable insights into the complex interplay of various factors that shape residents’ intentions to consume PMDs products. The factors are classified into three main categories, including internal reasons, external reasons, and consumer motives.

The external reasons would affect consumer motives directly. When people make PMDs purchasing decisions, they would be influenced by their social environment, such as the opinions of relatives and friends, media, and the influence of celebrities. Additionally, participants’ consumer motives are also influenced by product attributes such as price, brand, ease of purchase and cooking, packaging, storage, and hygiene. These results are consistent with previous studies [2,29,51,52,53]. According to the respondents, they tend to purchase PMDs out of impulse because of others’ buying behavior or feedback. Media can also generate attraction to them. They are especially attracted by live-streaming sales, which have become popular in China in recent years. Additionally, PMDs’ product features are equally important to the consumers. They will make purchase decisions by taking into account the potential risks and values that the products may bring. And, their attitudes may change with the product’s features. The results showed that external reasons have a significant positive impact on consumer motives.

Further, global motives have an effect on intention. As seen in the participants’ recordings, support from important others, positive attitude, and perceived value would boost PMDs consumption. On the contrary, opposition from important others, negative attitude, and perceived risk would make residents opt out of the purchase. Combining the research findings above, the results are consistent with behavioral reasoning theory (BRT) [47]. BRT classifies subjective norm, attitude, and perceived control as global motives, which are relatively broad substantive factors that consistently influence intention. To summarize, external reasons directly affect consumer motives, and motives have a direct impact on intention. In addition to the direct influence on intention, motives also serve as mediator variables.

In addition to these findings, the results show that internal reasons play a moderating role in the association between external reasons and consumer motives. In other words, regardless of how positive or attractive external factors may be, residents may not necessarily generate consumer motives. Previous studies have found that individual factors, such as self-assessed cooling skills, personal aspirations, personal roles, and interests, play a role in food choice decisions [54]. These findings highlight the importance of food skills, cooking skills, and attitudes toward home cooking, which can be understood as possible mechanisms explaining the consumption of PMDs [54,55,56]. The results of this study reveal that subject characteristics moderate the relationship between external reasons and consumer motives, which indicates that even if products have good quality and positive word-of-mouth, people with cooking-skill confidence and interest may still lack desire to purchase PMDs.

## 5. Conclusions and Future Recommendations

In conclusion, this study uses grounded theory to explore the determinants of the intention to consume PMDs and constructs the theoretical model of the influencing factors of PMDs’ consumer intention. The results revealed that external reasons are the main determinants directly influencing consumer motives, and have an indirect effect on consumer intention through consumer motives. Consumer motives directly affect consumer intention. In addition, internal reasons moderate the effect of external reasons on consumer intention, namely that external reasons cannot inspire consumer motives under the moderation of the internal reasons. It is the first time that all of these components are incorporated into one theoretical model. The model helps to summarize and understand not only the different factors that influence willingness to consume, but also how they interact with each other. Moreover, the government can carry out corresponding regulations and proper incentives to the market based on research results.

The advantage of the model is that it analyzes the data collected from 51 participants from diverse socio-economic backgrounds. This study restores the residents’ willingness to consume PMDs from a practical point of view. However, this study has the following shortcomings. First, although this study has been carefully analyzed based on grounded theory and with the support of Nvivo12 Plus, the construction and coding of the theoretical model remain subjective and have deviations due to the involvement of researchers. Future studies can expand the data collection and recruit more researchers to analyze the interview recordings to construct a more precise model. Second, in contrast to quantitative analysis, qualitative analysis can be conducted with a small sample size. However, it is essential to use a quantitative method to confirm the findings. Therefore, the determinants of residents’ intention to consume PMDs should be further discussed by quantitative research with large samples.

## Figures and Tables

**Table 1:** Demographic characteristics of residents (N = 51).

Characteristic	n	%	Characteristic	n	%
Gender			Age		
Male	19	37	18–24	6	12
Female	32	63	25–34	22	43
Marital status			35–44	7	14
Married	18	35	45–54	13	25
Unmarried	33	65	55 and above	3	6
Education level:			Career		
Less than high school	6	12	Student	8	16
High school/Secondary	4	8	Professional and technical staff	13	25
Junior college	6	11	Employees of enterprises or public institutions/public servant	14	27
Bachelor’s degree	20	39	Social production service workers/life service workers	6	12
Postgraduate	15	30	Unclassifiable person	10	20

**Table 2:** Open-coding results.

Initial Category	Original Concept
A1 Cooking skills	a1 the level of cooking skills; a2 ability to prepare food; and a3 common sense to cook
A2 Food skills	a4 ability to shop and select ingredients; a5 ability to match different kinds of food; and a6 ability to read product labels
A3 Cooking chores	a7 number of household members who can cook; a8 the length of cooking; a9 cooking experience; and a10 female cooking at home
A4 Attitudes toward daily diet	a11 personal attitudes toward cooking; a12 pursuing fashion trends; and a13 foodie
A5 Food quality	a14 ingredient list and nutrition labeling; a15 product ingredients and food material; a16 the net content of food; a17 edible method; a18 storage method; a19 date of production; a20 taste and flavor of production; and a21 sell-by date
A6 External value	a22 brand perception; a23 packaging appeal; a24 price; and a25 diversity and uniqueness of product
A7 Media campaign	a26 online advertising; a27 offline marketing
A8 Word-of-mouth	a28 product evaluation; a29 product sales
A9 Functional value	a30 save time; a31 save energy; a32 easy to preserve; a33 standardized taste of finished dishes; a34 no seasonal or regional restrictions; and a35 more affordable than takeout and restaurants
A10 Emotional value	a36 relaxing; a37 enjoyable
A11 Social value	a38 have face
A12 Cognitive value	a39 satisfy curiosity or taste; a40 for a change in flavor or to satisfy a craving
A13 Functional risk	a41 cannot satisfy all the family’s tastes; a42 hard to cook; a43 single flavor; a44 cannot replace a proper meal; a45 few varieties; and a46 poor quality of food
A14 Physical risk	a47 need to control diet; a48 low nutritional value; a49 harmful to human health; a50 unbalanced meat–vegetable ratio; a51 unhygienic; and a52 unknown source of food material
A15 Economic risk	a53 more expensive than home cooking
A16 Psychological risk	a54 disappointing; a55 worried
A17 Group effects	a56 purchased by people around; a57 social reaction; and a58 online group purchase
A18 Normative obedience	a59 resistance from family elders; a60 be submissive
A19 Cognitive	a61 knowledge of varieties, brands, and other information about PMDs; a62 the time of the first purchase
A20 Affective	a63 it is good; a64 refuse to purchase; a65 indifferent to PMDs; and a66 no need to purchase PMDs
A21 Willingness to purchase	a67 willing to purchase; a68 take a wait-and-see attitude; a69 unwilling to purchase; and a70 recommend others to purchase

**Table 3:** The categorization process of axial coding.

Main Category	Sub-Category		Initial Category	Relationship Connotation
Internal reasons	Subject characteristics		A1 Cooking skills	Cooking skills are a range of abilities to produce dishes with the help of physical means or mechanical equipment (e.g., cutting and stewing).
			A2 Food skills	Food skills are the ability to select and prepare ingredients and food material based on the knowledge and skills necessary for preparing needs-based meals (e.g., shopping and balancing diet).
			A3 Cooking chores	This refers to the individual daily household chore related to cooking.
			A4 Attitudes to a daily diet	This refers to the individual daily dietary perceptions.
External reasons	Product features		A5 Food internal quality	Inherent food characteristics of PMDs, including safety, health, reliability, convenience, etc.
			A6 Food external value	Non-inherent food characteristics of PMDs, including uniqueness, diversity, marketability, etc.
	Environmental features		A7 Media campaign	Publicize PMDs through online and offline means, such as social media platforms, TV, posters, etc.
			A8 Word-of-mouth	Discussions and information related to the brand, products, services on the web, etc.
Consumer motives	Perceived control	Perceived value	A9 Functional value	Perceived utility derived from the convenience and utility provided by product selection.
			A10 Emotional value	Perceptual utility derived from the sensation or emotion evoked by the choice of a product.
			A11 Social value	Perceived utility derived from associating with one or more social groups through product choice. It may lead to higher self-enjoyment, such as an increase in self-confidence and a sense of recognition by others.
			A12 Cognitive value	Perceptual utility resulting from the satisfaction of curiosity or novelty induced by the choice of a product.
		Perceived risk	A13 Functional risk	The likelihood that the products may be unfit for consumption, or may not function as expected.
			A14 Physical risk	The likelihood that the product will cause harm or pose a potential threat to an individual’s physical health.
			A15 Economic risk	The likelihood of monetary losses arising from the choice of a product.
			A16 Psychological risk	The likelihood of mental stress due to the choice of a product.
	Subjective norm		A17 Group effects	Influence on individuals from other individuals’ behaviors and attitudes in the social reference group.
			A18 Normative obedience	Individuals anticipate expectations from significant others or groups about whether they should perform a particular behavior, and individuals’ intentions about whether they are willing to conform to the expectations held by others.
	Attitude		A19 Cognitive	Cognition is a rational evaluation and an objective evaluation and awareness of a product based on the beliefs held by individuals about products’ attributes.
			A20 Affective	Emotion is a perceptual evaluation of a product based on an individual’s feelings and values.
Intention	Consumer intention		A21 Willingness to purchase	Individuals’ psychological tendency to purchase PMDs products.

**Table 4:** Typical relationship structure of main categories.

Typical Relationship Structure	Relationship Structure Connotation
External reasons → Consumer motives	External reasons include the external environment and product characteristics, which directly affect the residents’ motives.
Consumer motives → Purchase intention	Consumer motives involve subjective norm, attitude, and perceived control, which directly influence residents’ purchase intention.
Internal reasons ↓ External reasons → Consumer motives	Subjective characteristics are internal reasons that influence the strength and direction of the relationship between external reasons and residents’ motives.
External reasons → Consumer motives → Purchase intention	Consumer motives act as the mediator between external reasons and purchase intention.

## Data Availability

The data presented in this study are available on request from the corresponding author.

## Appendix A

**Outline of In-Depth Interview**
Part 1: Personal information
The information including gender, age, career, and education level.
Part 2: Questions about home cooking
1. The way to purchase food material. Prompt: How to buy food material (online or offline)? How long does it take you to buy food material? How much does the family spend on the food materials each month?
2. Arrangements for household chores related to cooking. Prompt: Who usually cooks? What is the approximate amount of time spent on cooking-related chores in a day? The differences between mid-week and weekend meals
3. The skills related to home cooking. Prompt: Do you know food preparation method? Do you know how to cook? Do you have food-related knowledge?
4. The advantages and disadvantages of home cooking. Prompt: What is your attitude towards cooking at home?
Part 3: Questions about the pre-made dishes
1. The knowledge of the pre-made dishes. Prompt: Do you know the pre-made dishes? When or how did you acquire knowledge of the pre-made dishes? What do you know about it? What is your attitude towards the pre-made dishes?
2. The attitudes towards the pre-made dishes. Prompt: What do you think are the advantages of the pre-made dishes? What are the biggest barriers to purchasing it? Would you recommend others to buy it?
3. Planning and shopping for the pre-made dishes. Prompt: How often do you ever purchase the pre-made dishes? Whether it is a spontaneous behavior or influenced by external factors? What are the main considerations for purchasing it?
4. Other questions. Prompt: Whether people around you buy the pre-made dishes? What are their attitudes toward it? What do you think about the current development trend of the pre-made dishes industry? What are your comments or ideas for improvement in the pre-made dishes industry?
